# ADVANCING CULTURALLY INCLUSIVE RESEARCH AND TOOLS IN ALZHEIMER’S DISEASE AND RELATED DEMENTIAS (ADRD)

**DOI:** 10.1093/geroni/igae098.2965

**Published:** 2024-12-31

**Authors:** Maryam Abdel Magid, Jean Taylor, Anne Turner

**Affiliations:** University of Washington, Seattle, Washington, United States; University of Washington, Seattle, Washington, United States; University of Washington, Seattle, Washington, United States

## Abstract

**Background and Significance:**

Existing research underscores cultural norms’ influence on healthcare decisions but lacks depth in exploring their impact on dementia care transitions, critical for improving ADRD care outcomes. Research Aims include (1) Analyze interviews with dementia care providers and staff to better understand how cultural norms influence the decision-making processes for care transitions. (2) Explore methods used to facilitate inclusive and engaging discussions regarding care transitions.

**Methods:**

Nested in the NIA-funded Decision Making in Alzheimer’s Research (DMAR) study, we examined interviews with 18 providers and staff on dementia care transitions. Prioritizing inclusivity, we leveraged the Alzheimer’s Disease Research Center (ADRC) registry and community partnerships coupled with tailored methods including diversifying the team and targeted outreach efforts. We’re employing a novel framework to analyze dementia care transitions, integrating societal and cultural aspects for nuanced understanding. This approach accounts for intergenerational culture, cognition perception, and care expectations, evolving to capture cultural nuances. Qualitative findings will be enriched with quantitative sentiment analysis, validated manually for accuracy.

**Key findings:**

Novel framework will allow us to identify and analyze cultural norms affecting care transitions, as perceived by ADRD providers. Implications: The study’s findings advocate for inclusive decision-making tools in ADRD care to accommodate diverse patient preferences. Future steps involve applying this framework to patients and caregivers and integrating findings into diverse settings involved in care decision-making. Funding statement: Funded in part by NIA grant #R01 AG066957 and the NLM T15 training grant #5T155LM007442-22. Content responsibility lies with authors, not representing NIH views.

